# Comparison of Anomaly Detection Methods on Event-Based Vision Sensor Data in a High Noise Environment

**DOI:** 10.3390/s26082320

**Published:** 2026-04-09

**Authors:** Will Johnston, Anthony Franz, Shannon Young, Rachel Oliver, Zachry Theis, Brian McReynolds, Michael Dexter

**Affiliations:** 1 Department of Engineering Physics, Air Force Institute of Technology, 2950 Hobson Way, Fairborn, OH 45433, USA; 2 Center for Technical Intelligence Studies and Research, Air Force Institute of Technology, 2950 Hobson Way, Fairborn, OH 45433, USA; 3 Department of Aeronautics and Astronautics, Air Force Institute of Technology, 2950 Hobson Way, Fairborn, OH 45433, USA; 4 Space Vehicles Directorate, Air Force Research Laboratory, 3550 Aberdeen Ave SE, Albuquerque, NM 87117, USA; 5 Department of Physics and Meteorology, United States Air Force Academy, 2354 Fairchild Drive United States Air Force Academy, Colorado Springs, CO 80840, USA

**Keywords:** dynamic vision sensor, event-based vision sensor, neuromorphic camera, hyperspectral analysis, frequency analysis, anomaly detection, principal component background suppression, Mahalanobis distance detector distance detector, Reed–Xiaoli detector, complementary subspace detector, high noise environment

## Abstract

Event-based vision sensors (EVSs) provide unique frequency analysis opportunities due to their event data output and high temporal resolution. Anomaly detection methods used in hyperspectral analysis can be used on the event frequency spectra to detect targets. However, the introduction of a strong, flickering interfering source can reduce the EVS sensitivity and obscure targets of interest. Previous work presented a method showing that targets could still be detected through an overwhelming source using frequency analysis, background suppression, and statistical filtering. This paper extends that research and compares the ability of five different eigenanalysis anomaly detection methods (principal component background suppression (PCBS) with peak threshold detection, Mahalanobis distance (MD) detector, complementary subspace detector (CSD), Reed–Xiaoli (RX) detector, and subspace Reed–Xiaoli (SSRX) detector) to detect targets in a high noise environment. The PCBS, MD, and CSD detectors performed well and were able to detect the targets through the overwhelming source. The PCBS detector had the best performance at low false-alarm rates (a > 400% detection probability increase at a false-alarm probability of 10^−5^). While the MD and CSD detectors had the best detection at higher false-alarm probabilities (approximately 7 × 10^−2^), the MD detector had a sub-second execution time. Depending on the application, the PCBS or MD detector are the best choice out of these five methods to detect targets in this type of high noise environment.

## 1. Introduction

**Figure 1:**
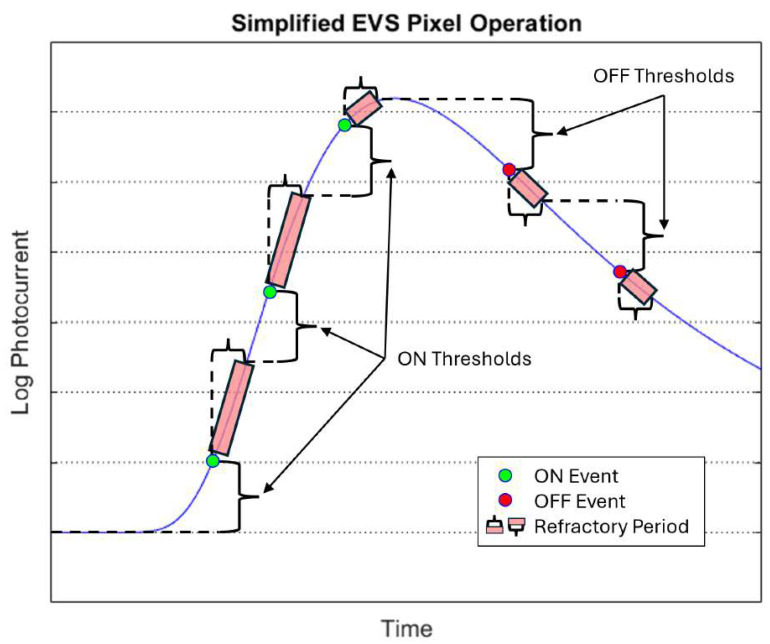
Simplified EVS pixel operation showing how events are generated based on the logarithm of the input intensity, tunable thresholds, and refractory period [1]. The blue line represents the logarithm of the of an EVS pixel’s photocurrent.

Event-based vision sensors (EVSs) are cameras that detect changes in scene brightness, have asynchronous pixel operation, and output an event stream at the pixel level instead of integrated frames like a traditional camera. Figure 1 depicts a simplified EVS pixel operation during event generation [1]. Events are generated when the logarithm of the induced photocurrent changes more than a set threshold. Once an event is generated in a pixel, it is combined with events from other pixels into a single event stream containing pixel location, timestamp, and event polarity (intensity increase or decrease). This is known as Address Event Representation (AER). These sensors deliver high temporal resolution (up to 1 µs), low latency (<1 ms), wide dynamic range, and low power consumption, while capturing dynamic information in a scene with sparse data requirements [2,3]. EVSs have demonstrated great potential in diverse applications—ranging from gesture recognition [4] and celestial object tracking [5,6] to particle detection [7,8] and visual navigation [9,10].

**Figure 2:**
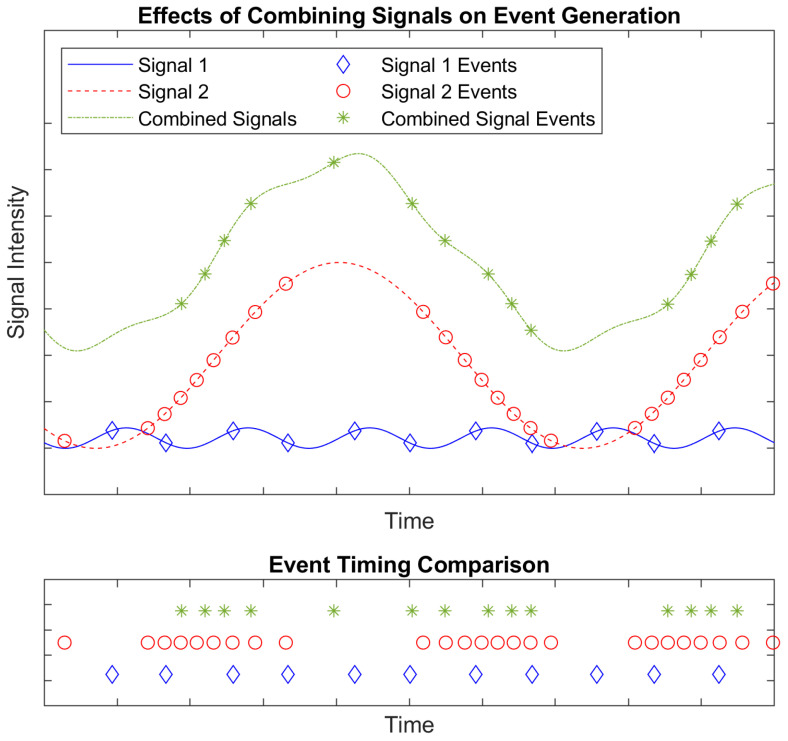
Approximated effects of adding signals on event generation using a rudimentary EVS model, showing a combined signals’ events will have timing characteristics that differ from the individual signals’ events. The signal intensity represents the photocurrent (or logarithmic photocurrent) in a pixel. The events in the timing comparison subplot are separated vertically for easier visualization. Adapted from [1].

In the presence of an overwhelming source creating a high noise (rapidly changing and bright) environment, integrating sensors suffer from saturation which reduces the ability to see features or targets of interest. EVS sensitivity and detection capability can also suffer from an overwhelming source, though not in the same way as a traditional camera. Because EVS pixels do not integrate photocurrent but rather respond to photocurrent changes in real time, saturation does not occur. However, features can become obscured due to reduced contrast when the background is changing. Figure 2 shows approximated effects of combining signals on pixel event generation using a rudimentary EVS model: both number of events and event timing are altered.

Extensive research has been conducted in several key areas similar to this problem, including dim target detection [11,12,13]; noise, background, and extraneous events reduction [14,15,16,17,18,19]; and dynamic occlusion removal [20,21]. However, this problem is unique in that the normal EVS noise and background reduction techniques are less effective due to the strong, continuous influence of the overwhelming source. Previous work proposed as a solution to this specific problem was a hardware change that subtracts the effects of the overwhelming source before event generation [22]. However, this center-surround method is currently only a proposed concept and no physical prototypes are available as of yet. Additionally, our previous work [1] presented a method showing that through event frequency analysis, background suppression, and statistical filtering, targets could still be detected in spite of this high noise environment [1].

This work extends our previous research and focuses on frequency-based anomaly detection methods to detect targets within this unique problem set using a novel sensing paradigm. This paper compares the newly developed anomaly detection method (principal component background suppression (PCBS) with peak threshold detection [1]) with common hyperspectral methods (Mahalanobis distance (MD) detector [23], complementary subspace detector (CSD) [24], Reed–Xiaoli (RX) detector [25], and subspace Reed–Xiaoli (SSRX) detector [26]) to determine initial best practices for a data-driven solution to detect targets in the presence of an overwhelming source. The five different anomaly detection methods were applied to 100 simulated scenarios containing a random number of targets (maximum of 30), with random frequencies, in the presence of an overwhelming source with a random power (chosen from four continuous sources) and a temporal profile generated by the atmospheric model. A fast Fourier transform (FFT) was applied to the simulated event data to generate the individual pixels’ frequency spectra for each scenario. Methods’ performances were compared using receiver operating characteristic (ROC) curves, a modified area under the curve metric, and specific false-alarm probabilities.

The PCBS, MD, and CSD detectors performed well and were able to detect the targets through the overwhelming source. The PCBS detector had the best general and low false-alarm rate performance. The MD detector had the best detection at higher false-alarm probabilities with a sub-second execution time. Overall, depending on the application, the PCBS or MD detector are the best choices out of these five methods to detect targets in this type of high noise environment.

This paper compares the newly developed PCBS method to standard hyperspectral anomaly detection methods for detecting targets in a high noise environment using EVS data. For the modeled scenarios, the PCBS method has the highest performance at low false-alarm probabilities, while the MD detector has slightly better performance at higher false-alarm probabilities and a lower computational requirement.

## 2. Materials and Methods

All analysis was performed using MATLAB 2023a on a personal computer containing an 11th Generation Intel Core i7-11800H at 2.50 GHz, 16 logical processors, and 64 GB of RAM. Computation time varied by scenario and detection method, as discussed later in this article.

### 2.1. Simulated Scenarios

ASSET (Air Force Institute of Technology Sensor and Scene Emulation Tool) was used to generate the simulated scenario frames, targets and overwhelming sources while v2e was used to transform those frames into event data. ASSET is a “physics-based image-chain model used to generate synthetic electro-optical and infrared (EO/IR) sensor data with realistic radiometric properties, noise characteristics, and sensor artifacts” [27,28]. The selection of ASSET was motivated by its strength in modeling atmospheric effects and sensor response. v2e is a Python and OpenCV code that “generates realistic synthetic [EVS] event [data] from intensity frames” at higher resolution [29].

**Figure 3:**
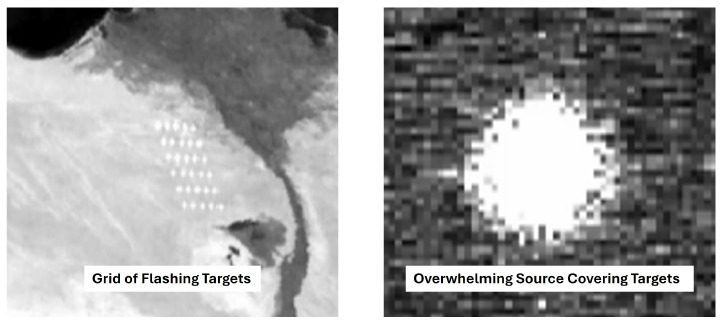
ASSET scene simulation image (zoomed in) showing the target grid without an overwhelming source (**left**) and the effects of an overwhelming source in the same scene as the targets (**right**) on a traditional sensor. Both images display the same FOV and viewpoint [1].

**Figure 4:**
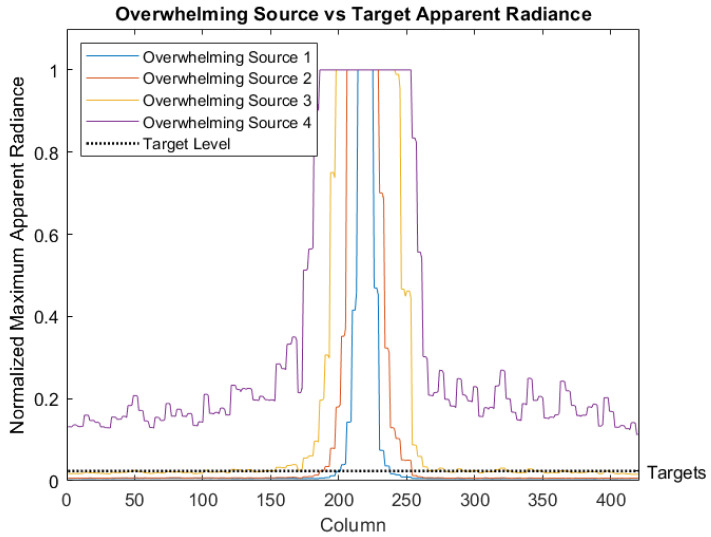
ASSET-modeled normalized apparent radiances of the four overwhelming sources, given as the maximum over a single FPA dimension. The target’s peak apparent radiance is shown for comparison, normalized to the same scale [1].

Simulated scenarios were developed using a methodology similar to that of our previous work [1], with some modifications. To generate a sample of 100 potential scenarios, a random number of flickering targets were placed at locations randomly chosen on a six-by-five grid. Target frequencies were randomly chosen from the set containing 3, 6, 8, 10, 12, and 17 Hz. To avoid bias in the aggregated results, all random selections were chosen from a uniform distribution. An upper frequency limit of 17 Hz was used because high-frequency targets generate more events and are, consequently, easier to detect. A lower frequency limit of 3 Hz was chosen to account for the possibility of undersampling the target behind the overwhelming source. The 10 Hz band was chosen to stress the methods’ detection capability as that frequency was the same as the average overwhelming source frequency. The other frequencies were chosen to sample frequencies between the bounds, while providing more low frequencies where detection will be more difficult. The targets’ flickering intensities were generated in ASSET by applying a sine wave to their power over time. All targets were modeled with the same signal-to-background radiance, ranging from one to approximately six over their temporal evolution. One of four continuous overwhelming source powers was chosen randomly for each scenario. Overwhelming sources’ frequencies were approximately 10 Hz (similar to the expected Greenwood frequency for the modeled scenario [30]) with stochastic apparent size variations, generated by the atmospheric model. The overwhelming source’s weakest power was chosen to make target detection relatively trivial through normal visualization techniques. The overwhelming source’s strongest power was chosen to be three orders of magnitude greater than the weakest, making visual detection nearly impossible for all targets. All scenarios used a 420 × 651 pixel focal plane array (FPA) and were simulated to be 5 s long with the ASSET simulation at 200 frames per second (FPS), which were then upsampled in v2e to 5000 FPS. Figure 3 shows zoomed-in images of the simulated target grid with and without an overwhelming source on a traditional sensor. Figure 4 shows the relative apparent intensity differences between the overwhelming sources and targets, as measured by a traditional sensor. These scenarios allow for a range of signal-to-background radiance ratios between approximately 0.0236 and 281 [1].

**Figure 5:**
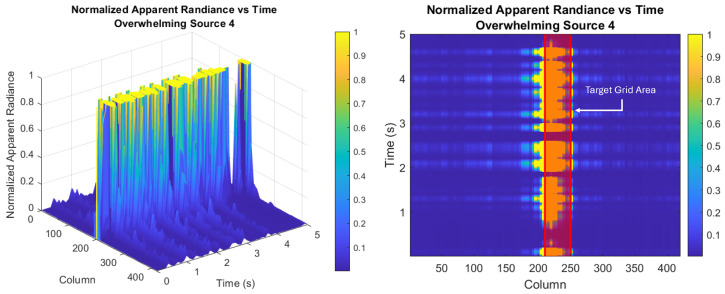
Temporal evolution of the apparent radiance for the strongest overwhelming source modeled along a pixel row passing through its center, as measured by a traditional sensor. The overwhelming source is modeled as a continuous source with apparent fluctuations due to the atmospheric propagation. The surface plot reiterates the high intensity of the overwhelming source and captures its dynamics over time, while the color plot indicates the location of the target grid area within those dynamics. In both plots, the “Column” axis refers to the pixels’ placement in the singular row. Adapted from [1].

Figure 5 shows the temporal evolution of the apparent radiance for the strongest overwhelming source along a pixel row passing through its center, as measured by a traditional sensor. The surface plot reiterates the high intensity of the overwhelming source and captures its dynamics, while the color plot indicates the location of the target grid area within those dynamics. In both plots, the “Column” axis refers to the pixels’ placement in the singular row.

### 2.2. Fast Fourier Transform on Event Data

**Figure 6:**
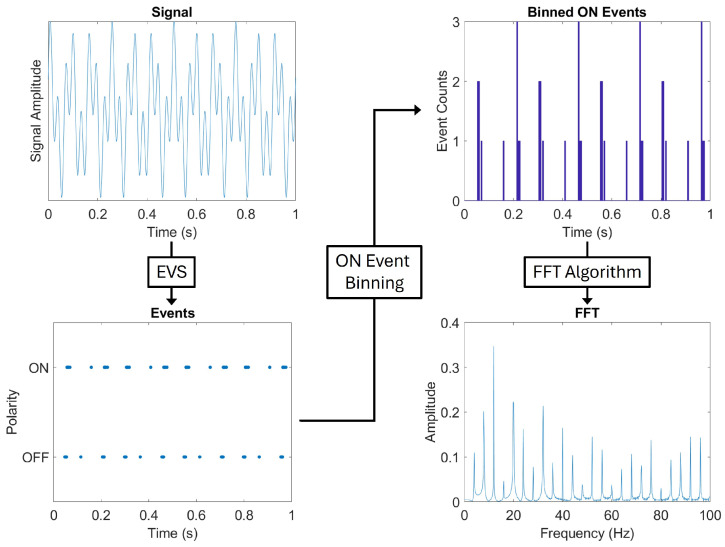
Flow chart depicting the process of transforming a signal to event data, and event data to a frequency spectra by summing ON events into time bins (5 ms for this example) and applying an FFT algorithm. Adapted from [1].

Event data must first be modified before an FFT can be applied due to the non-uniform time intervals between generated events. For this work, events were separated by pixel address and summed into 5 ms time bins. Only ON events were used as it was observed that OFF events display a decay pattern, introducing extraneous frequency information. Previous work demonstrated that using both ON and OFF events introduces more noise, resulting in an order of magnitude higher false-alarm rate during detection [1]. Figure 6 depicts the process of turning a signal into events (v2e) before binning and applying an FFT [1]. Once the pixel frequency spectra were generated, the anomaly detection methods could be applied.

### 2.3. Anomaly Detection Methods

Anomaly detection refers to the process of identifying targets when there is no knowledge about the target. The detection decision is based solely on the difference from an assumed or estimated background model. With the exception of the newly developed method, these anomaly detection techniques are derived from the generalized likelihood ratio test by manipulating the Mahalanobis distance and employing different background models and assumptions. While there are more anomaly detection methods, these five methods make the most simple assumptions and require less to be known about the scene. Requiring less to be known about the scene is important when studying methods to be applied to scenarios with lots of unknown factors.

#### 2.3.1. Principal Component Background Suppression with Peak Threshold Detection

The PCBS with peak threshold detection method assumes that a frequency spectra matrix, **X**, contains both the signal, **S**, and background, **B**, such that,

(1) X=S+B.

Additionally, the method assumes the frequency spectra’s eigenvector matrix, V, can be decomposed as

(2) $$V=\left[\hat{B}\;\hat{S}\right],$$

where $\hat{B}$ and $\hat{S}$ are estimated background and signal eigensubspaces, respectively. The background eigenspace is assumed to be some number of the largest eigenvectors, also known as principal components (PCs), because the PCs with the largest eigenvalues capture the most variance across the pixels. Since the targets are rare, the background drives the variance and the patterns in the background spectra are captured in the large eigenvalue modes. The appropriate number of PCs to include in the background eigenspace was determined by identifying when subtracting an additional PC starts to have little effect on the maximum value frequency spectrum. The maximum value frequency spectrum is the maximum amplitude of each frequency across all pixels. This diminishing return point was found using an angle-based elbow method on the maximum value frequency spectra difference curve created by summing the differences of each frequency amplitude between consecutive PC-subtracted maximum value frequency spectra [1]. The proper division of the eigenspace is an active area of research and most methods tend to use arbitrary thresholds. A complete evaluation of the stability and reliability of the elbow method used in this work is beyond the scope of this paper. The elbow method was chosen because it adapts to each scenario to find the bend and identify the point of diminishing returns. For consistency, the elbow method was applied to all scenarios.

The estimated signal in the data space can then be calculated by either subtracting the background projected data or by simply projecting the data onto the signal, such that,

(3) $$\tilde{S}=X-\tilde{B}=X-\hat{B}{\hat{B}}^{T}X=\hat{S}{\hat{S}}^{T}X,$$

where $\tilde{B}$ and $\tilde{S}$ are the estimated background and signal models in the data space [1].

To identify the target pixels, a peak prominence of three standard deviations was used to first identify significant frequencies. A threshold was then applied to the amplitudes of those frequencies, individually, to identify target pixels. This threshold was scaled to produce the ROC curves, similar to thresholding the detection statistics in the following hyperspectral anomaly detection methods. It is important to note that the identified frequencies may not be same as the target frequencies due to phase differences with the overwhelming source and FFT aliasing and spectral leakage. However, this is inconsequential as the method simply seeks to identify pixels with statistically significant frequency content after the background suppression. The PCBS method, as well as the other methods analyzed in this work, identifies anomalies from trends in the spectral patterns. If the previously mentioned issues exist, they are consistent and will either be removed with the background or characteristic of the targets’ spectra [1].

#### 2.3.2. Mahalanobis Distance Detector

The MD detector assumes the background is normally distributed and the clutter is spatially invariant. This detector is often employed due to its simplicity and speed, even if the true background is not normally distributed. Poor performance of the MD detector can be an indication that the background does not fit a normal distribution. The MD is a measurement between a data vector, x, and the mean data vector, $\hat{\mu }$, relative to the standard deviation of the background distribution in the direction of $\hat{\mu }$ to x. The detection statistic, $r_{MD}$, is given by

(4) $$r_{MD}\left(x\right)={\left(x-\hat{\mu }\right)}^{T}{\hat{\Sigma }}^{-1}\left(x-\hat{\mu }\right),$$

where x is the pixel frequency spectrum, $\hat{\mu }$ is the mean pixel frequency spectrum, and $\hat{\Sigma }$ is the frequency spectra standard covariance matrix given by

(5) $$\hat{\Sigma }=\frac{{\left(X-\hat{\mu }\right)}^{T}\left(X-\hat{\mu }\right)}{n-1},$$

where *n* is the number of pixels. Diagonalizing the covariance matrix such that,

(6) $$\hat{\Sigma }=\hat{V}\hat{D}{\hat{V}}^{T},$$

where $\hat{V}$ is the eigenvector matrix and $\hat{D}$ is the diagonal eigenvalue matrix, the MD can be rewritten as

(7) $$r_{MD}\left(x\right)={\left[{\hat{D}}^{-1/2}{\hat{V}}^{T}\left(x-\hat{\mu }\right)\right]}^{T}\left[{\hat{D}}^{-1/2}{\hat{V}}^{T}\left(x-\hat{\mu }\right)\right].$$

Using the whitened form of the data,

(8) $$z={\hat{D}}^{-1/2}{\hat{V}}^{T}\left(x-\hat{\mu }\right),$$

the MD reduces to

(9) $$r_{MD}\left(z\right)={\left|\left|z\right|\right|}^{2}.$$

in the eigenspace. A threshold can then be applied to the detection statistics to identify anomalous spectra and target pixels [23,31].

#### 2.3.3. Complementary Subspace Detector

Similar to PCBS, the CSD detector assumes the frequency spectra’s eigenvector matrix can be decomposed into two separate subspaces, estimating the background and signal eigenmodels. This detector applies an MD detector to these subspaces before taking their difference. The detection statistic, $r_{CSD}$, is given by

(10) $$r_{CSD}\left(z\right)=\left|\right|{\hat{S}}^{T}{z||}^{2}-\left|\right|{\hat{B}}^{T}z{\left|\right|}^{2}$$

in the eigenspace [24,31]. The eigensubspaces can be determined using the same elbow method described in Section 2.3.1.

#### 2.3.4. Reed–Xiaoli Detector

**Figure 7:**
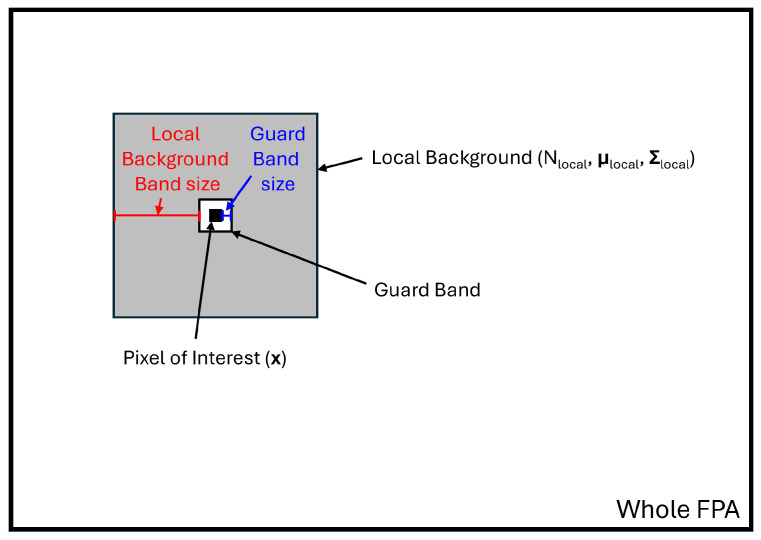
Example depiction of an RX detector pixel of interest, guard band, and local background. Adapted from [31].

The RX detector assumes the scene has structured clutter that contains varied background statistics based on location in the scene. This detector applies an adaptive MD detector that narrows the background statistics to a local background surrounding the pixel of interest instead of using the global statistics. A guard band was used to avoid including potential signal in the background statistics, as shown in Figure 7. The RX detector involves a trade-off where too small of a background provides a poorly conditioned covariance matrix and too large of a background negates the adaptability of the method. The general rule is to make the local pixel background sample size greater than the square of the number of frequency bands ($N_{local}>K^{2}$). To assist with the matrix inversion issue a quasi-local matrix is used instead of a fully local covariance matrix. The detection statistic, $r_{RX}$, is given by

(11) $$r_{RX}\left(x\right)={\left(x-{\hat{\mu }}_{local}\right)}^{T}\hat{V}{\hat{D}}_{local}^{-1}{\hat{V}}^{T}\left(x-{\hat{\mu }}_{local}\right),$$

where ${\hat{\mu }}_{local}$ is the local mean spectrum, $\hat{V}$ is the global eigenvector matrix, and ${\hat{D}}_{local}^{-1}$ is the local eigenvalue matrix. This detection statistic can be given in the PC space as

(12) $$r_{RX}\left(z\right)={\left(z-{\hat{\mu }}_{local}\right)}^{T}{\hat{D}}_{local}^{-1}\left(z-{\hat{\mu }}_{local}\right),$$

where $z=x\hat{V}$ [25,31]. However, due to the high frame rate of the modeled EVS and length of the simulated recording, the recommended number of local background pixels was 250,000 (K=500 frequencies), almost the size of the focal plane array (FPA) used in this work. Furthermore, execution time for the RX was on the order of an hour per scenario while the previously discussed methods could be computed in seconds. To improve the condition of the local covariance matrices and the computation time, the spectra were downsampled to integer frequencies through summation and reduced to an upper limit of 50 Hz. A two-pixel guard band and a 25-pixel background band were used for the RX detector. A guard band is needed to prevent any target data from contaminating the local background statistics. A two-pixel guard band was chosen as it was large enough to remove target blurring effects from the almost single-pixel targets. A 25-pixel background was chosen to balance the need to maintain a small local background with the need to capture a statistically relevant sample size within the limitation of the 420 × 651 FPA pixel count.

#### 2.3.5. Subspace Reed–Xiaoli Detector

The SSRX detector is applied similarly to the RX detector by accounting for the local statistics; however, it subtracts an estimated global background eigensubspace to eliminate whole-frame clutter (i.e., the MD detector applied using the local background statistics while subtracting the global background). The detection statistic is given in the eigenspace by

(13) $$r_{SSRX}\left(z\right)={\left(P_{B}^{⊥}z-P_{B}^{⊥}{\hat{\mu }}_{local}\right)}^{T}{\hat{D}}_{local}^{-1}\left(P_{B}^{⊥}z-P_{B}^{⊥}{\hat{\mu }}_{local}\right),$$

where the local mean is in the eigenspace and the operator $P_{B}^{⊥}$ is given by

(14) $$P_{B}^{⊥}=I-\hat{B}{\hat{B}}^{T}.$$

Similar to the PCBS and CSD detectors, $\hat{B}$ is an estimated background eigensubspace containing some number of the largest eigenvectors [26,31]. The background eigenspace can be determined using the same elbow method described in Section 2.3.1.

Like the RX detector, the SSRX suffers from a long execution time and an ill-conditioned covariance matrix with these scenarios. Downsampled data sets, a two-pixel guard band, and a 25-pixel background band were used for the SSRX detector as well.

### 2.4. Detection Method Analysis

For each method, detection statistic thresholds were scaled to generate empirical ROC curves. ROC curves represent detection and false-alarm probabilities, $P_{D}$ and $P_{FA}$, over all possible detection statistic thresholds and are used to compare performance between classification methods. The detection probability is given by,

(15) $$P_{D}=\frac{\mathrm{True}\;\mathrm{Positive}\;\mathrm{Detects}}{\mathrm{Total}\;\mathrm{Targets}},$$

while the false-alarm probability is calculated as,

(16) $$P_{FA}=\frac{\mathrm{False}\;\mathrm{Positive}\;\mathrm{Detects}}{\mathrm{Total}\;\mathrm{Pixels}}.$$

This micro-averaging method is common for aggregating results of a detection method applied to applications where scenarios have different numbers of targets and characteristics [32]. Additionally, scenario variables were selected randomly, within the constraints, from a uniform distribution to avoid bias in the aggregated results, as discussed in Section 2.1. Detection success was determined by assuming a single-pixel target located at the center of the target. A one-pixel guard band was incorporated for false-alarm probability calculations as to not include target bloom in false positives. The more the ROC curve is pushed to the upper left limit of a unit square, the better the method’s performance [33]. To create ROC curves that were more general to each method and less scenario-specific, detection probabilities at the same false-alarm probabilities were combined by adding the number of true positives in each scenario and divided by the total number of targets across all scenarios.

Uncertainty in the detection probability is given by the standard approximate confidence interval,

(17) $$\epsilon_{P_{D}}=\pm z_{\alpha /2}\sqrt{\frac{P_{D}\left(1-P_{D}\right)}{N}},$$

where $z_{\alpha /2}$ is the inverse of the standard normal cumulative distribution function evaluated at a probability of 1−α and *N* is the number of trials [34]. A 95% confidence interval was used for the uncertainty.

To simplify the ROC curves for quantitative comparison, they were reduced to a single performance metric using the area under the curve (AUC). The AUC is given by,

(18) $$\mathrm{AUC}=\int_{0}^{1}P_{D}\left(P_{FA}\right)\;d\left(P_{FA}\right),$$

where the detection probability is a function of the false-alarm probability. The closer the AUC is to one, the closer the ROC curve is to perfect (unit square, $P_{D}\left(P_{FA}\right)=1\;\forall \;P_{FA}$) and the better the method’s average performance [33]. However, the AUC is biased toward higher false-alarm probabilities and provides little distinction between ROC curves that have a steep and early increase in detection probability. Logarithmically compressing the false-alarm probability shifts the bias to the left, allowing for a better comparison in those cases. Additionally, if targets are sparse on a large FPA, a large number of false alarms will drown out the small number of targets. Normalizing the logarithmically compressed area by the logarithm of the total number of pixels scales the upper limit to one again but allows for more emphasis on lower false-alarm probabilities. The normalized logarithmic area (logAUC) is given by,

(19) $$\mathrm{logAUC}=\frac{1}{\log_{10}\left(A_{FPA}\right)}\int_{\log_{10}\left(A_{ROI}^{-1}\right)}^{0}P_{D}\left(\log_{10}\left(P_{FA}\right)\right)\;d\left(\log_{10}\left(P_{FA}\right)\right),$$

where $A_{FPA}$ is the area of the FPA in pixels. The uncertainty in the logAUC is given by propagating the uncertainty in the detection probability through the trapezoidal rule,

(20) $$\epsilon_{\mathrm{logAUC}}=\sqrt{\frac{1}{4}\sum_{i=1}^{I-1}{\left(\log_{10}\left(P_{FA,i+1}\right)-\log_{10}\left(P_{FA,i}\right)\right)}^{2}\left(\epsilon_{P_{D},i+1}^{2}+\epsilon_{P_{D},i}^{2}\right)}.$$

While the calculation allows for a simplification of the ROC curve to a single metric, the logAUC is still representative of general performances and may not correctly capture potential unacceptable false-alarm probabilities for different applications. Therefore, both the logAUC and specific false-alarm probabilities spanning $10^{-4}$ to $10^{-2}$ were used as performance metrics.

## 3. Results

**Figure 8:**
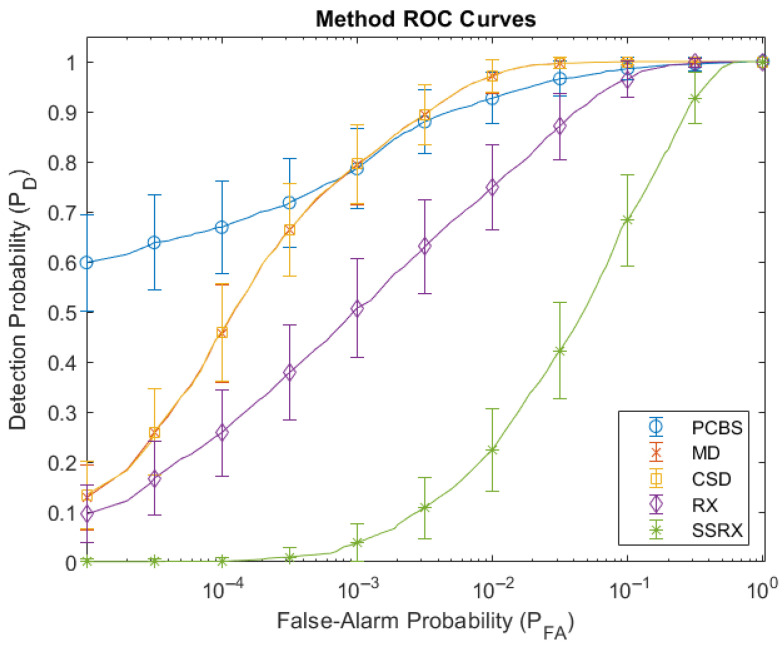
Methods’ ROC curves with standard approximate 95% confidence intervals.

**Figure 9:**
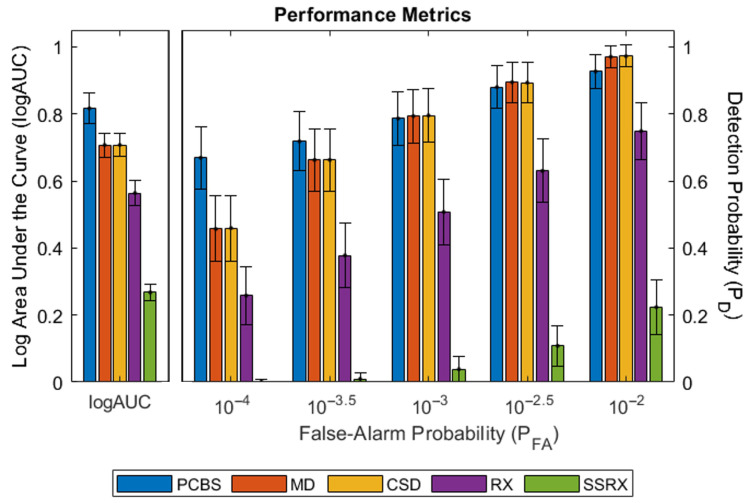
Methods’ performance metrics with standard approximate 95% confidence interval. The leftmost grouped bars show the logAUC for each method and the right five grouped bars display the detection probabilities at specific false-alarm probabilities.

Figure 8 displays the combined ROC curves for each method. Figure 9 shows the performance metrics where the leftmost grouped bars represent the logAUC for each method and the right five grouped bars display the detection probabilities at specific false-alarm probabilities. The PCBS detector performed better than the other four methods at low false-alarm probabilities (a > 400% average detection probability increase at a false-alarm probability of 10^−5^, approximately three false-alarm detections across the 273,420 pixel FPA). A low false-alarm probability is important for accurate target/background discrimination. The MD and CSD detectors had almost equal results. They also performed slightly better at higher false-alarm probabilities than the PCBS detector. Due to the design of the automatic background eigenspace determination method in the PCBS and CSD detectors, they had a slightly slower computation time than the MD detector (approximately 15 s versus sub-second, respectively). The RX and SSRX detectors’ performances were poor at all false-alarm probabilities with run times exceeding multiple minutes even with a downsampled data set.

**Figure 10:**
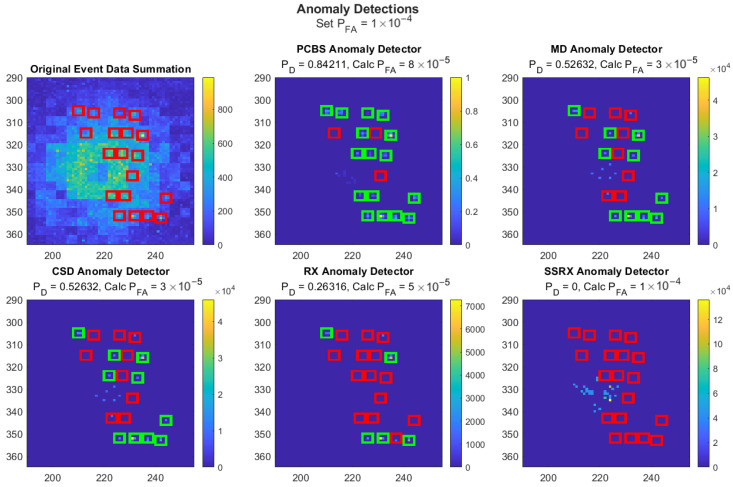
Example scenario, zoomed in to aid visualization, using each anomaly detection method at a false-alarm probability of $10^{-4}$. The green boxes indicate detected targets (true positives), the red boxes indicate undetected targets (false negatives), and the other highlighted pixels are false-alarms (false positives).

Figure 10 shows an example scenario, zoomed in to aid visualization, using each anomaly detection method at a false-alarm probability of $10^{-4}$. The green boxes indicate detected targets (true positives) and the red boxes indicate undetected targets (false negatives). The other highlighted, but not boxed, pixels are false-alarms (false positives). The calculated false-alarm probability was less than the set/desired false-alarm probability with some methods because many pixels can have the same detection statistic, causing discrete false-alarm probabilities and potentially relatively large jumps with a single threshold shift.

It is important to note that the three targets not detected by the PCBS detector in the example scenario have a frequency of 3 Hz and are close to the overwhelming source. This result is as expected since lower frequency targets are more undersampled due to the overwhelming source. This result was verified by the presence of a detected 3 Hz target in the upper right corner of the grid. This target was detected because it is farther away and less affected by the overwhelming source.

## 4. Discussion

The PCBS, MD, and CSD detectors performed well when detecting targets in this high noise environment using EVS data. While different applications could have a higher tolerance for false-alarms, the PCBS detector had the best low false-alarm probability performance (a > 400% detection probability increase at a false-alarm probability of 10^−5^, approximately three false-alarm detections across the 273,420 pixel focal plane array) with a computation time of approximately 15 s. If the application allows for higher false-alarm probabilities, the MD detector could be the better choice due to its sub-second execution time and high detection at higher false-alarm probabilities (approximately 7 × 10^−2^). These three detectors had the best detection performance most likely due to the overwhelming source creating a large global background. These detectors either remove this background through eigensubspace subtraction and/or detect anomalies from the global mean and variance. The PCBS detector most likely had the highest performance at lower false-alarm probabilities because it has additional filtering processes that target significant frequency content.

Poor performance, a run time of minutes per scenario, and the requirement to downsample the frequency spectra to achieve that test-reasonable run time create practical limitations for real-time application. For these reasons, we ruled out the RX and SSRX detectors as viable methods in these types of scenarios. The unacceptable run times of these detectors for these scenarios is caused by the need to recalculate the local statistics for each pixel of interest, five orders of magnitude more calculations than the other methods. This poor RX and SSRX performance is most likely due to using a small local background that is more sensitive to small deviations and makes the covariance matrix more unstable in an environment where the overwhelming source generates a more global background.

Future work includes characterizing the detection limits, as targets near the center of the overwhelming source and with lower frequency were more difficult to detect. These limits can be characterized as a metric of apparent power on the sensor by taking into account several entangled factors: background radiance (a brighter background will generate a larger initial photocurrent), target and overwhelming source radiance ranges (a high minimum radiance or a smaller range will generate less events) and frequencies (lower frequencies may be undersampled), length of the recording (longer recordings allow for more opportunity to accurately sample the targets), and distance of the target from the overwhelming source (effects produced by the overwhelming source weaken with distance from its center). Additionally, future work includes a comparison to high-speed standard cameras, validating the simulated data set results through laboratory data collection, improving the methods using other frequency analysis techniques (e.g., non-uniform FFTs or interspike intervals) or Machine/Deep Learning algorithms, testing different target frequency profiles, and testing the detectors in a similar way on simulated moving targets as movement will add another layer of difficulty.

## 5. Conclusions

When comparing five anomaly detection methods (principal component background suppression, Mahalanobis distance, complimentary subspace, Reed–Xiaoli, and subspace Reed–Xiaoli detectors) in a high noise environment using EVS data, the PCBS, MD, and CSD detectors performed well and were able to detect the targets through the high noise generated by the overwhelming source. The PCBS detector had the best performance at low false-alarm rates (a > 400% detection probability increase at a false-alarm probability of 10^−5^). While the MD and CSD detectors had the best detection at higher false-alarm probabilities (approximately 7 × 10^−2^), the MD detector had a sub-second execution time. Depending on the application, the PCBS or MD detector are the best choice out of these five methods to detect targets in this type of high noise environment caused by an overwhelming source.

## Data Availability

Data underlying the results presented in this paper are not publicly available at this time but may be obtained from the authors upon reasonable request.

## Abbreviations

The following abbreviations are used in this manuscript:

EVS	event-based vision sensor
AER	address event representation
FFT	fast Fourier transform
PCBS	principal component background suppression
MD	Mahalanobis distance
CSD	complementary subspace detector
RX	Reed-Xiaoli
SSRX	subspace Reed-Xiaoli
ASSET	Air Force Institute of Technology Sensor and Scene Emulation Tool
EO	electro-optical
IR	infrared
PC	principal component
FPS	frames per second
FPA	focal plane array
ROI	region of interest
ROC	receiver operating characteristic
AUC	area under the curve
logAUC	logarithmically compressed area under the curve
